# Intubation-free in vivo imaging of the tracheal mucosa using two-photon microscopy

**DOI:** 10.1038/s41598-017-00769-6

**Published:** 2017-04-06

**Authors:** Tibor Z. Veres, Tamás Kopcsányi, Marko Tirri, Armin Braun, Masayuki Miyasaka, Ronald N. Germain, Sirpa Jalkanen, Marko Salmi

**Affiliations:** 1 MediCity Research Laboratory, University of Turku, Turku, 20520 Finland; 2 Institute of Biomedicine, Turku Center for Disease Modeling, and Turku PET Centre, University of Turku, Turku, 20520 Finland; 3 Department of Preclinical Pharmacology and In Vitro Toxicology, Fraunhofer Institute for Toxicology and Experimental Medicine, and Institute of Immunology, MHH; Hannover, 30625 Germany; Biomedical Research in Endstage and Obstructive Lung Disease Hannover (BREATH), Member of the German Centre for Lung Research (DZL), Hannover, Germany; 4 WPI Immunology Frontier Research Center, Osaka University, Osaka, 565-0871 Japan; 5 Lymphocyte Biology Section, Laboratory of Systems Biology, National Institute of Allergy and Infectious Diseases, National Institutes of Health, Bethesda, MD 20892 USA; 6 Department of Medical Microbiology and Immunology, University of Turku, Turku, 20520 Finland

## Abstract

The mucosal layer of conducting airways is the primary tissue exposed to inhaled microorganisms, allergens and pollutants. We developed an *in vivo* two-photon microscopic approach that allows performing dynamic imaging studies in the mouse trachea, which is a commonly used *in vivo* model of human small-diameter bronchi. By providing stabilized access to the tracheal mucosa without intubation, our setup uniquely allows dynamic *in vivo* imaging of mucociliary clearance and steady-state immune cell behavior within the complex airway mucosal tissue.

## Introduction

During the last decade, multi-photon intravital microscopy (MP-IVM) has become the “gold standard” technique for imaging biological processes *in vivo* and in real time at single-cell resolution^1^. The variety of applications ranges from visualizing brain activity^2^ and cancer cell spread^3^ to deciphering how cellular interaction dynamics shape the host response to immunological insults^4^. The main advantage of this imaging modality is that it allows us to see deep below the surface of tissues (typically 200–600 µm, depending on the organ), which permitted direct observation of how cells behave in a complex tissue environment. This has been especially important in case of the immune system, since the motility of its cells throughout the body is a key determinant of its normal function^5^.

While immune dynamics within most lymphoid and non-lymphoid organs has been successfully addressed in great detail, imaging the airways and lungs has always been difficult due to (1) movement artifacts generated by breathing, (2) air-filled alveoli restricting optical penetration and (3) deep localization requiring invasive surgery. While most of these technical obstacles have been overcome, MP-IVM has only allowed to observe the most peripheral alveoli beneath the pleural wall^6^. However, several inflammatory diseases, e.g. asthma and viral bronchitis, develop in the walls of the conducting airways, which are located deep beneath the lung surface. In addition, many immunologically or toxicologically relevant agents are inhaled as droplets or particles (agglomerates) with preferential or partial impact in the conducting airways. Fungal spores, allergens like pollen, and pollutants like silica particles or asbestos fibers often occur in a particulate form with sizes typically ranging between 3–100 µm. Likewise, viruses and bacteria are often inhaled as droplets or agglomerates with a similar size distribution. Thus, all of these infectious or toxic materials preferentially impact on the mucosal layer of the bronchi^7^, making this the primary location for biological responses. Since the mucosal layer of the intrapulmonary airways is largely inaccessible for *in vivo* imaging, several groups^8–10^, including us^11, 12^, have established the trachea as an appropriate model to visualize airway mucosal immune responses using MP-IVM. It has also been suggested earlier that the mouse trachea is a highly relevant model of human small bronchi due to its similar architecture and physiology^8, 13^.

Previous studies achieved immobilization of the trachea for imaging using an intubation cannula^8, 9, 11^. However, an endotracheal tube will alter the normal physiology of tracheal mucosal tissue due to mechanical irritation. In humans, even a short-term endotracheal intubation can cause significant tracheal inflammation^14^. It will also interfere with the function of the mucociliary escalator, since the physiological air-liquid interface that would allow the free upward flow of mucus is not present. Here we present an improved method without intubation that provides stabilized access to the tracheal mucosa for observation via MP-IVM. The new approach helps to minimize inflammation and vascular leakage, preserves steady-state immune cell behavior and allows direct observation of physiological mucociliary clearance. This new technique will allow the *in vivo* monitoring of physiological, toxicological and immune responses to a wide range of inhaled agents in the naïve trachea *in vivo*.

## Results

### Basic engineering, design and functionality of the intubation-free imaging setup

**Figure 1:**
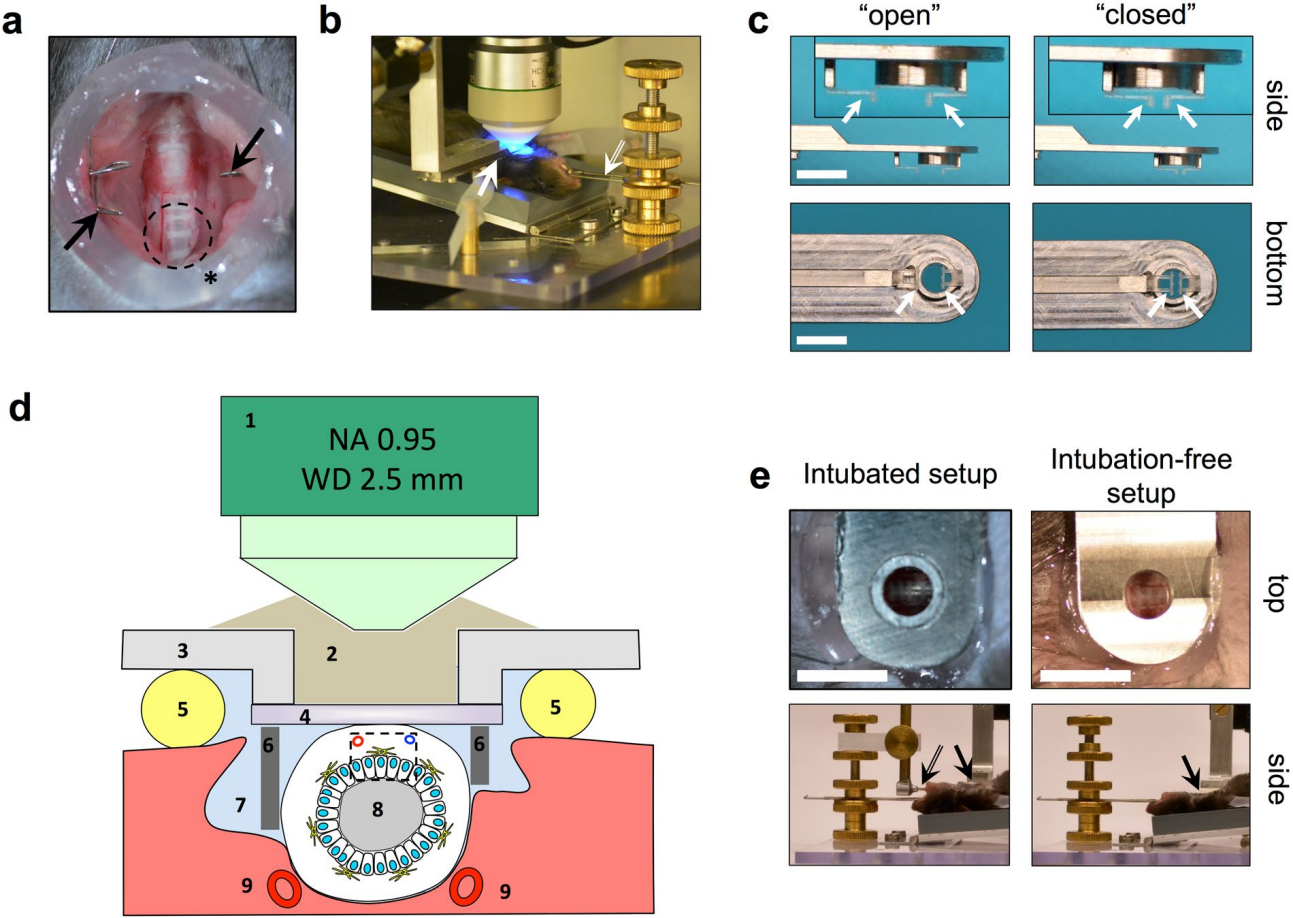
Technical setup of intravital microscopy on the mouse trachea. (**a**) Trachea of an anesthetized mouse after micro-surgical exposure. Arrows indicate the wire retractor. Asterisk indicates vacuum grease ring. Dashed circle indicates the typical observation area. (**b**) Imaging setup with a custom-made stage. Arrow indicates the custom-made “tracheal window”. Double arrow indicates the tooth bar (see Supplementary Figure 1 for details). (**c**) Improved, adjustable “tracheal window” for imaging the intubation-free trachea. Images show the two end-positions (“open” and “closed”) of the adjustable mechanism that allows fine adjustments of the 3D-printed plates (arrows) that provide external, lateral support of the trachea. Inserts in top images show the adjustable supporters at higher magnification. Bar = 5 mm. (**d**) Schematic diagram (non-proportionate) showing the cross-section of the trachea with the basic principle of accessing it for observation via MP-IVM. Numbers indicate: long working-distance objective lens (1); immersion medium (2); frame of imaging window (3); coverslip (4); vacuum grease (5) for sealing the observation area; lateral support mechanism (6) for the intubation-free setup; peritracheal space filled up with saline (7); tracheal lumen (8); carotid arteries (9). Dashed box indicates the volume typically accessed for imaging. (**e**) Tracheal preparation ready for imaging experiment (intubated and intubation-free setup). Upper panels show the observation area as seen through the “tracheal window” in top-view (bar = 5 mm); lower panels show in side-view the relation of the intubation cannula (double arrow) and other parts of the stage to the tracheal window (arrows).

We previously created an intravital microscopy stage that provides optical access to the micro-surgically exposed trachea (Fig. 1a) in anesthetized, spontaneously breathing mice (Fig. 1b, see also Supplementary Figure 1). In the original setup, an endotracheal tube is used to stabilize the trachea for imaging, and a custom-designed “imaging window” is positioned on the trachea with high precision^12^. Given that this intubated setup is not ideal for the preservation of physiological conditions in the tracheal mucosa, we improved this method by developing a new window that provides stabilized access to the trachea without intubation (Fig. 1c). We achieved this end by designing a support mechanism that can be accurately adjusted to provide the necessary lateral support to immobilize the trachea, without compressing the tissue (Fig. 1d,e). During the design process, we were aided by micro-CT to better understand the *in situ* anatomy of the surgically exposed trachea (Supplementary Figure 2). 3D-modeling in combination with Computer Numeric Control (CNC) precision milling or 3D-printing (Supplementary Figure 3) were used for the rapid production of prototypes including very small parts with well-defined specific shapes and dimensions.

### Maintenance of physiological conditions at the tracheal mucosa via intubation-free imaging

**Figure 2:**
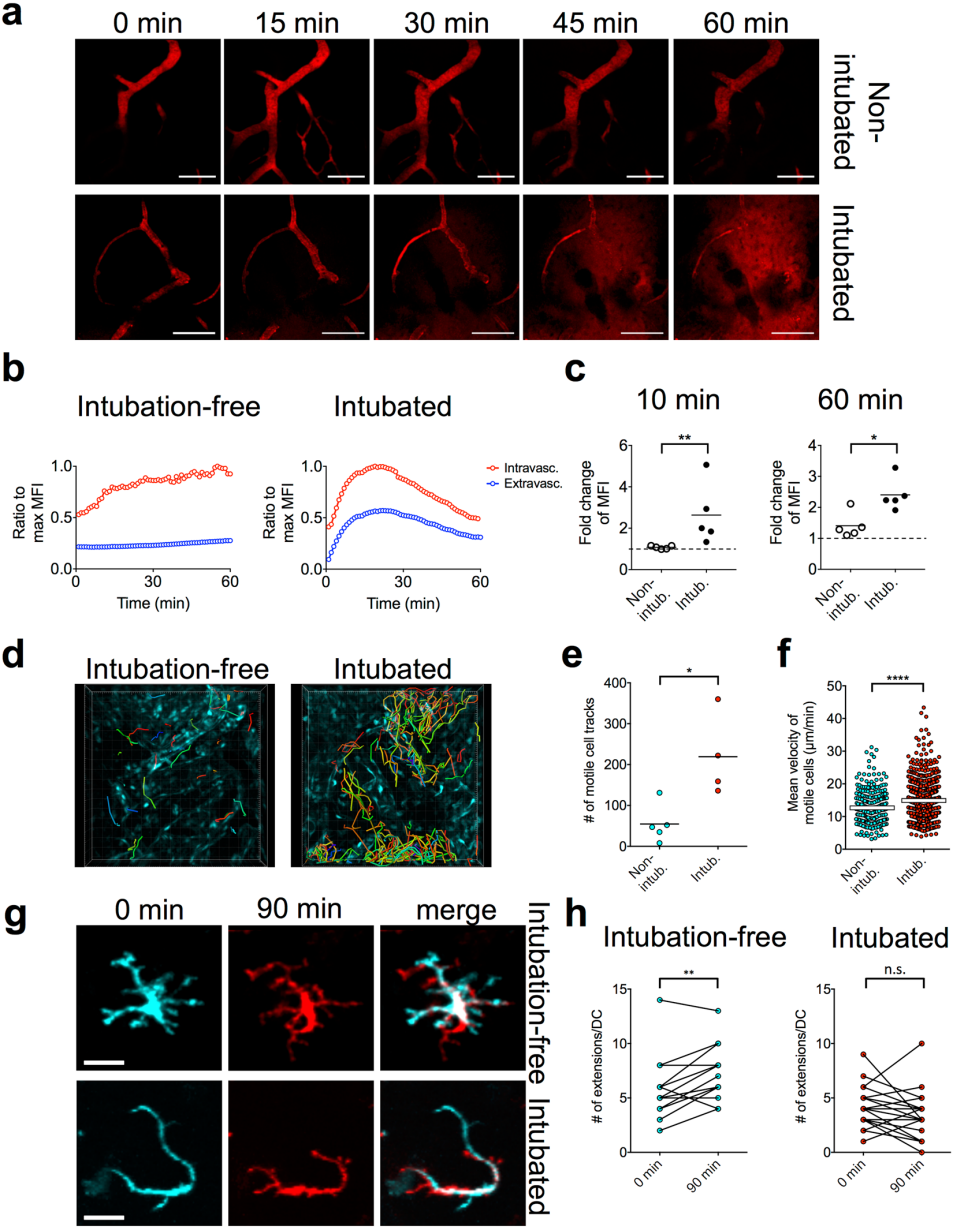
Comparison between the intubated and intubation-free setup. (**a**–**c**) Analysis of vascular leakage by i.v. injected fluorescent dextran, 155 kDa for (**a**); 2,000 kDa for (**b** and **c**). (**a**) Snapshots from Supplementary Videos 1 and 3 are shown, bar = 100 µm. (**b**) Changes of MFI in the intravascular vs. extravascular compartment during a 60 min imaging period. Curves are representative of 5 measurements performed with each setup. (**c**) Analysis of changes in MFI in the extravascular compartment at 10 min or 60 min after dextran injection. MFI of the extravascular space at 1 min was used as a reference (dashed line represents value of 1 = no change; *p < 0.05; **p < 0.01 as determined using Mann-Whitney test, n = 5 mice). (**d**–**f**) Analysis of inflammatory cell recruitment to the interstitial space using ubiquitously fluorescent CAG-ECFP mice. (**d**) Snapshots from Supplementary Videos 1 and 3, colored lines indicate tracks of motile cells, grid spacing = 20 µm. (**e**) Number of motile cell tracks within a defined volume of tissue (346 µm × 346 µm × 33 µm), detected during a period of 60 min. Cells with a minimum speed of >2 µm/min were considered as motile (*p < 0.05; as determined using Mann-Whitney test, n = 4–5 mice). (**f**) Analysis of mean velocities; each dot represents one cell. Data were pooled from the analysis of n = 4–5 mice (****p < 0.0001 as determined using Mann-Whitney test). (**g**,**h**) Analysis of IE-DC dynamic probing behavior. (**g**) Snapshots of individual IE-DCs at the beginning and at the end of a 90 min imaging period (with overlay, see also the corresponding Supplementary Videos 6 and 7), bar = 20 µm. (**h**) Number of dendritic extensions/IE-DC; each dot represents an IE-DC. The same cell was analyzed at the beginning and at the end of a 90 min imaging period (**p < 0.01 and n.s.: non-significant, as determined using paired t-test).

Since vascular leakage and neutrophil accumulation are the hallmarks of a tissue response to irritation and injury^15, 16^, we first measured to what extent our original and intubation-free setups affect these processes in the tracheal mucosa. We analyzed vascular permeability by i.v. injection of fluorescent dextran and observation of the blood vessels and the surrounding interstitial space of the trachea (Fig. 2a). In the intubation-free setup, fluorescence intensity in the extravascular areas remained low during an observation period of 60 min (Supplementary Videos 1 and 2), whereas intubation caused a rapid and extensive leakage of dextran (Supplementary Video 3), detectable as rapidly as 10 min after injection. Although at 60 min we observed some small leakage in the intubation-free setup as well, this was clearly below the values we measured after intubation (Fig. 2b,c). To detect neutrophil accumulation, we used ubiquitously fluorescent CAG-ECFP or CAG-DsRed mice and looked for the accumulation of highly motile cells around blood vessels. As we expected, there were only a few motile cells around blood vessels in the intubation-free setup (Supplementary Videos 1 and 2), whereas intubation caused the rapid accumulation of leukocytes, presumably neutrophils, inside the vessels, which then rapidly extravasated and infiltrated the surrounding tissue (Supplementary Video 3). Quantitatively, we defined motile cells as having a minimum speed exceeding 2 µm/min and followed their tracks during the imaging period (Fig. 2d). We found that both the total number of the cell tracks (Fig. 2e) and the average speed of the detected cells (Fig. 2f) were higher in case of the intubated setup. Collectively, these results suggest that mechanical irritation by the endotracheal tube causes significant inflammation over time, which can be avoided by the preferential use of the new method.

It has been suggested that inflammatory signals profoundly affect the activity of dendritic cells at peripheral sites^17^. Therefore next we asked whether the intubation-induced irritation causes changes to the normal behavior of intraepithelial dendritic cells (IE-DCs). IE-DCs are strategically located in the tracheal epithelium and serve as sentinels of the respiratory mucosal immune system^18^. They show a typical “Langerhans-cell-like” morphology with several extensions (Supplementary Figure 4). It has been suggested that they use these extensions to “probe” inhaled antigens by projecting them across the epithelium into the airway lumen^19^. When we observed IE-DCs using CD11c-EYFP reporter mice in intubation-free settings, they constantly formed new, highly motile, branching extensions in all directions (Supplementary Video 4). The number of extensions remained constant or increased during a 90 min imaging period (Fig. 2g,h). However, when imaged using tracheal intubation, IE-DCs appeared less active and they had fewer extensions. Over time, IE-DCs showed abnormally elongated extensions that were mostly immotile (Supplementary Video 5) or “pulled back” their few extensions and finally became “rounded-up” cells (Fig. 2g,h and Supplementary Videos 6 and 7). This suggests that the intubation-induced inflammation alters the function of resident IE-DC populations, which can be avoided by using the new procedure.

### Visualizing steady-state IE-DC probing activity in the complex airway mucosal tissue

**Figure 3:**
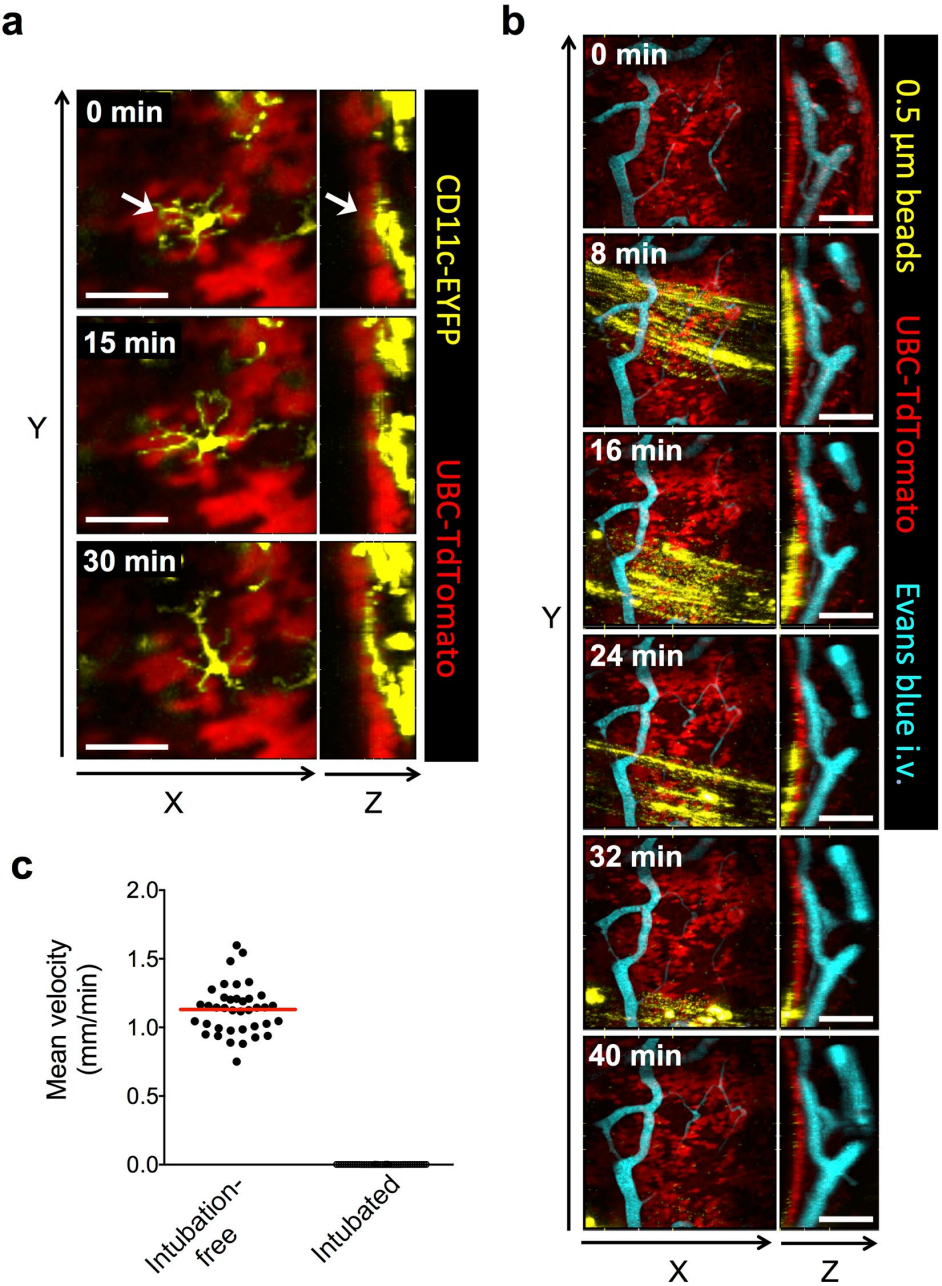
Visualizing IE-DC probing behavior and mucociliary clearance in the steady-state. (**a**) IE-DCs were visualized in CD11c-EYFP x UBC-TdTomato mice using the intubation-free setup. Snapshots from Supplementary Video 9 are shown as XY and ZY representations, bar = 50 µm. Arrows indicate an IE-DC. (**b**) Yellow/green fluorescent 0.5 µm polystyrene beads were o.ph. delivered to UBC-TdTomato mice and the tracheal mucosa was visualized using the intubation-free setup. Evans blue was injected i.v. via a tail vein catheter to visualize blood vessels. Snapshots from Supplementary Video 10 are shown as XY and ZY representations, bar = 100 µm. (**c**) The mean velocities of bead aggregates with an approximate size of 10 µm were determined from single-plane video-rate movies (such as the one shown in Supplementary Video 12). Each dot represents the velocity of a single bead aggregate; horizontal red line indicates mean value. Data were pooled from the analysis of 2 mice.

Having established an intubation-free approach that preserves physiological conditions of the airway mucosa, we next explored the power of this setup to visualize IE-DC probing in the context of the surrounding complex tissue. To this end, we crossed CD11c-EYFP and UBC-TdTomato mice to examine the *in situ* behavior of IE-DCs in relation to the epithelium. In universal reporter systems, such as UBC-TdTomato mice, the tracheal epithelium is readily identifiable by its intensive red fluorescence^12^. In rapidly fixed tracheal tissue, MHC-II^+^ IE-DCs show a characteristic morphology with multiple branching extensions, resembling Langerhans cells of the skin (Supplementary Figure 4). Since we observed earlier with the intubation-free setup that IE-DCs over time continuously re-create this prototypical morphology, we assumed that their motility revealed by MP-IVM would reflect their steady-state behavior. When individual IE-DCs were carefully examined (Fig. 3a and Supplementary Videos 8 and 9), we found that their “probing” activity was directed in a lateral direction and they did not reach the airway lumen (i.e. no trans-epithelial extensions are formed), suggesting that in the steady-state, IE-DC protrusions do not penetrate the airway epithelium.

### Visualizing the mucociliary clearance of instilled particles

Since the intubation-free setup ensured that the internal environment of the trachea remained intact, we anticipated that it would allow us also to visualize the mucociliary clearance of oropharyngeally (o.ph.) instilled beads in real time. To test this idea, we delivered 0.5 µm green fluorescent beads by the o.ph. route to UBC-TdTomato mice and followed the fate of the beads via intubation-free tracheal MP-IVM. Shortly after administration, we observed the rapid transport of beads on the surface of the epithelium (Fig. 3b and Supplementary Video 10). Of note, the beads were completely immotile when using the intubated setup (Supplementary Video 11). Surprisingly, the beads were not homogenously distributed on the entire mucosal surface, but instead formed discrete “rafts” of beads and bead aggregates rapidly flowing upwards, while the rafts themselves also showed a slow movement in the lateral direction during the imaging period. The bead “rafts” usually passed by only once during the first 60 min of image acquisition, after which we could not observe them again. Next we measured the speed of bead transport. To this end, we acquired single-plane, video-rate sequences (Supplementary Videos 12 and 13), since the beads moved too rapidly to be accurately followed by serial Z-stack acquisition. Although we were able to detect signal from individual beads, these moved rapidly in-and-out of the focal plane. Therefore we followed larger (**~**10 µm) bead aggregates to measure the overall speed of mucociliary transport; this was about 1.1 mm/min in the steady-state. As a comparison, beads were immotile when the intubated setup was used (Fig. 3c). These data confirm that avoiding intubation is absolutely essential in order to observe mucociliary clearance *in vivo*.

## Discussion

Here we describe an improved, less invasive approach to perform MP-IVM on the mouse trachea and provide evidence that avoiding endotracheal intubation is essential in order to maintain naïve physiological conditions of the airway mucosa (a detailed comparison of the intubated vs. intubation-free setup is shown in Table 1). Moreover, we demonstrate that intubation-free imaging uniquely allows dynamic observation of airway mucociliary clearance. This improved system opens up several new avenues for future studies on the airway mucosa in both health and disease. Since this method enables investigations in naïve, non-inflamed tissue, it will also allow the monitoring of relatively small effects that are induced by inhalation of toxic or pro-inflammatory agents. This might be of particular interest for studying viral respiratory infections, allergic reactions in response to allergens like house dust mite or toxicological effects of inhaled particles or mixtures like diesel exhaust.

**Table 1:** Comparison of the new, intubation-free setup with the previously used, intubated approach.

	Intubation-free setup	Intubated setup
Advantages	• Reduced inflammation	• Better stability
	• Reduced vascular leakage	
	• Preserved DC motility	• Easier access to the trachea
	• Intact mucociliary elevator function	• Better signal due to shorter distance from coverslip window
Disadvantages	• Image stability not always optimal	• Pronounced inflammation and vascular leakage
	• Optical access to the epithelium can be more difficult	• Disturbed DC physiology
		• Disrupted mucociliary escalator function

In humans, the mucosa of the conducting airways represents an important anatomical/physiological barrier to the outside environment. This barrier function is maintained by the airway surface liquid (ASL) containing mucus; the ciliated, pseudostratified epithelium; and by the strategically localized IE-DC network. Clearance of inhaled particles via the mucociliary escalator is essential to protect us from potentially dangerous microorganisms, allergens and environmental pollutants; failure to do so, such as in cystic fibrosis, can cause severe disease. In inflammatory disorders affecting the small airways, such as allergic asthma, physiological and structural changes in the airway epithelium and subepithelial tissue (“airway remodeling”) may cause pathological broncho-obstruction^20^, with local immune responses organized by resident DCs playing an important role in the development of such changes^19^. It has been suggested earlier that IE-DCs initiate immune responses against inhaled antigens by capturing them via transepithelial extensions (“periscope function”)^21^. However, our dynamic *in vivo* observations of IE-DC motility in the steady state did not reveal the formation of such extensions; we only observed them occasionally in a previously used *ex vivo* system^11^. A recent study employing dynamic imaging on explanted tracheas also suggested that DC projections do not cross the epithelium^22^. The lack of transepithelial DC extensions in the steady-state would make sense, since an adaptive immune response should only target pathogens that breach the epithelial barrier. However, a compromised epithelial barrier might allow IE-DCs to mediate sensitization against innocuous antigens, as observed in asthma^23^. Our model will certainly allow a more detailed *in vivo* imaging analysis on how DC-epithelial interactions contribute to airway disease in both allergy and infection.

Airway mucociliary clearance is severely impaired in asthma, chronic obstructive pulmonary disease (COPD) and cystic fibrosis^24^. This process has been assessed *in vivo* in both humans^25^ and animals^26^ using various radioisotope-based techniques. However, direct microscopic observations have been done only using *in vitro* air-liquid interface cultures^27^ or *ex vivo* explanted tracheas^28^. In both cases, the ciliated epithelium is submerged in buffer solution. This is very different from the *in vivo* situation, in which a thin layer of mucus-containing fluid film is moved upwards by the beating cilia of epithelial cells. Several groups have measured the “normal” speed of particle transport using such explanted, submerged tracheas. The reported velocities showed a large variation between 10–200 µm/s (corresponding to 0.6–12.0 mm/min) among studies using slightly different setups; these values depended on the distance from the epithelial surface where the measurement was performed and the size of the beads used^28–30^. However, to our knowledge, mucociliary particle transport has not been visualized/measured earlier *in vivo*, using such a minimally invasive method as presented here. Therefore, we believe that our intubation-free MP-IVM approach will offer a completely new “modality” in this respect and will become a widely used technique to study physiological processes like mucociliary clearance and steady state immune cell behavior and probing movements of IE-DCs in murine airways. In addition, the technique can be used to study pathophysiological mechanisms in mouse models of airway diseases like asthma, COPD, lung infection and cystic fibrosis. Considering the enormous number of genetically modified mouse models (many of them with specific functional or cell-specific fluorescent labels) this technique will enable a wide range of mechanistic studies in airway research.

## Methods

### Animals

CD11c-EYFP mice were a kind gift from Dr. Michel C. Nussenzweig (The Rockefeller University, NY); UBC-TdTomato mice were generated at the Lymphocyte Biology Section, Laboratory of Systems Biology, NIAID/NIH. CAG-ECFP and CAG-DsRed mice were purchased from The Jackson Laboratory. For MP-IVM experiments, female mice were used at 12–20 weeks of age. Animals were maintained at the Central Animal Laboratory of the University of Turku. All animal procedures were approved by the Ethical Committee for Animal Experimentation in Finland. They were done in adherence with the rules and regulations of the Finnish Act on Animal Experimentation (62/2006) and were performed according to the 3R-principle (animal license number 5588/04.10.07/2014).

### Microscopy stage design

Our custom-made microscopy stage was originally designed and built in the fine mechanical workshop of Fraunhofer ITEM, Hannover, Germany; additional improvements were done in the fine mechanical workshop of the University of Turku. Individual functional parts, such as levers for holding the limbs, the base plate with adjustable angle, the device with screws for holding the tooth bar/intubation cannula and the coarse manipulator (UM-3C, Narishige) were all mounted on an acrylic glass plate (Supplementary Figure 1).

### Tracheal window design

The improved window for imaging on the intubation-free trachea was designed using SketchUp Make (Ver. 15.3.329); moving parts were engineered using Solid Edge ST8. Individual stainless steel parts were produced via CNC milling (Feinmechanik Zimmermann GmbH). Lateral supporting parts (Supplementary Figure 3e,f) were designed using SketchUp Make and produced by 3D printing using Visijet-X printing material (Multiprint 3D Oy).

### Tracheal instillation

0.5 µm Yellow/Green or Red FluoSpheres (Life Technologies) were diluted 1:20 and instilled in a volume of 20 µl via the oropharyngeal (o.ph.) route under isoflurane anesthesia. Animals were allowed to recover before inducing surgical anesthesia.

### Tracheal microsurgery

Mice were anesthetized using 75 mg/kg Ketamine and 1 mg/kg Medetomidine i.p. The ventral neck area was shaved and a 30 G cannula, connected to a piece of PE 10 polyethylene tubing (Becton Dickinson) was inserted into the tail vein for the i.v. administration of vascular labels during imaging. Mice were then placed on our custom-made microscopy stage (Supplementary Figure 1) in a supine position. The front teeth were hooked into the tooth bar; the limbs were slightly stretched and attached to the stage using duct tape. A small piece of skin was removed from the ventral neck area and the trachea was exposed via careful blunt dissection. The pre-tracheal muscles were pulled to the side and kept apart using a retractor (hand-made using 0.2 mm piano wire). The connective tissue covering the trachea was carefully dissected, a “ring” of vacuum grease was applied on the edge of the surgical area (using a 10 ml syringe) and the whole area was filled up with PBS using a pipette. A drop of PBS was applied on the bottom side of the tracheal window, which was then moved into position above the trachea. The imaging window was then carefully moved towards the tracheal surface (using the coarse manipulator) until the coverslip touched the trachea and the trachea was very gently squeezed from the side by the 3D-printed lateral supporting parts until movements of the trachea were completely eliminated. Finally, a drop of Immersol W immersion medium (Carl Zeiss) was applied on the imaging window, into which the microscope objective was dipped. During the entire imaging analysis, the microscope stage with the intravital preparation and the microscope’s nosepiece were enclosed in a dark chamber and kept at a temperature of 34 °C, which is close to the mouse’ thermoneutral zone. The preparation was allowed to equilibrate with the chamber environment for 20 min before acquisition of images. Immediately before image acquisition, vascular labels (155 kDa or 2,000 kDa Fluorescent Dextrans from Sigma-Aldrich; or Qtracker 655 Vascular Label from Life Technologies) were injected via the tail catheter.

### Image acquisition

Multidimensional (XYZT and up to 4 colors) images were acquired using a Leica SP5 MP system equipped with a Chameleon Vision II and a Chameleon Ultra II femtosecond laser (Coherent), using excitation at 865 nm (for ECFP and TRITC) or between 920–950 nm (for Yellow/Green or red FluoSpheres, EYFP, TdTomato and Evans blue). Emission filters were 483/32 (for ECFP), 525/50 (for Yellow/Green FluoSpheres) 535/50 (for EYFP), 585/40 (for TdTomato and Red FluoSpheres) and 650/50 (for Evans blue and Qtracker 655). All imaging was done with an HCX IRAPO L 25×/0.95 W objective with a WD = 2.5 mm. Using this specific objective was essential, since the level difference between the top surface of the imaging window and the tracheal wall was >2 mm. 3D imaging of DC probing behavior and mucociliary clearance of beads was performed at an XYZ resolution of 256 × 256 × 60 with 1.6x zoom factor. These settings provided the best compromise between XY resolution, Z resolution and time resolution. Stacks were repeated at every 40–60 s, for a duration of 60–90 min, after which movement artifacts or decrease of signal intensity impeded further image acquisition.

### Visualization and image analysis

Imaris 8.1.2 was used for visualization (generation of axial projections and “extended section” views) and for quantitation of cell motility (via automated spot detection). Quantitative analysis of vascular leakage was done as described earlier^31^. The number of IE-DC extensions was counted manually; the total number of terminal branches was counted for each cell. Annotation of videos was done using ImageJ Fiji^32^; display of multiple videos (or separate channels) in the same frame was achieved using QuickTime Pro 7.7.8.

### Micro-CT

Axial micro-CT image series of the micro-surgically exposed trachea were done using a Siemens Inveon Multimodality PET-CT imaging scanner. A standard CT setup with low-dose upgrade was used in “rat mode” configuration. The following parameters were used in the scans: CT X-ray tube voltage 80 kV, 500 uA anode current, high geometrical magnification. Gantry rotation was done with discrete steps during scanning; 441 projection images were taken using a total rotation of 220°. The projected size of acquired images was 1536 × 1024 pixels using binning 2, trans-axial and axial, respectively. In the reconstructed CT image, using down sampling factor 2, the final pixel size was 0.041 mm in X Y and Z directions. The reconstructed image had a resolution of 768 pixels in X and Y directions, and 512 pixels in Z direction.

## Electronic supplementary material

Supplementary information

Supplementary Video 1

Supplementary Video 2

Supplementary Video 3

Supplementary Video 4

Supplementary Video 5

Supplementary Video 6

Supplementary Video 7

Supplementary Video 8

Supplementary Video 9

Supplementary Video 10

Supplementary Video 11

Supplementary Video 12

Supplementary Video 13
