# Supplementary material for: Intubation-free in vivo imaging of the tracheal mucosa using two-photon microscopy
Source: Sci Rep. 2017 Apr 6;7:694. doi: 10.1038/s41598-017-00769-6 (PMC5429620; doi:10.1038/s41598-017-00769-6)
Supplement: Supplementary file 1 — Supplementary information [file 41598_2017_769_MOESM1_ESM.pdf]

Supplementary Information

Intubation-free in vivo imaging of the tracheal mucosa using two-photon microscopy

Tibor Z. Veres, Tamás Kopcsányi, Marko Tirri, Armin Braun, Masayuki Miyasaka, Ronald N. Germain, Sirpa Jalkanen and Marko Salmi

Supplementary Figure 1

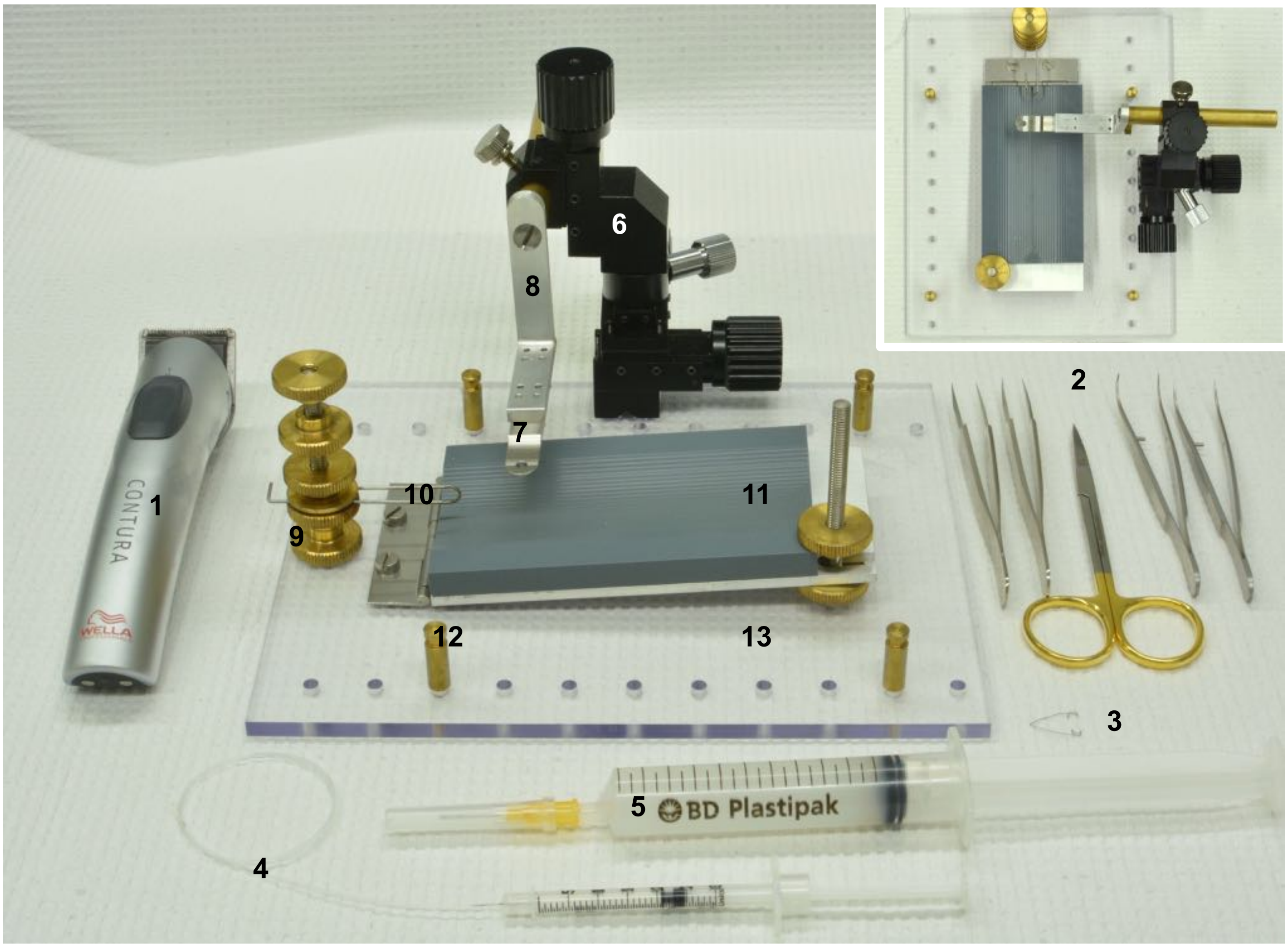

Supplementary Figure 1: Custom-made microscopy stage and equipment for in vivo tracheal imaging. Clipper (1) for shaving the ventral neck area; surgical equipment (2) including scissors for skin incision and tweezers for blunt dissection of the trachea; custom-made retractor (3) to expose the trachea; cathether and syringe (4) for i.v. access; syringe with vacuum grease (5) for sealing the exposed area; coarse manipulator (6) for accurate positioning of the imaging window (7) attached to a holder device (8); device with screws (9) for holding the tooth bar (10) or the intubation cannula (not present); base plate with adjustable angle (11) for positioning the mouse; lever with multiple positions (12) for holding the limbs using tape; acrylic base plate of the stage (13). Insert in the top right corner shows the stage in top-view.

Supplementary Figure 2

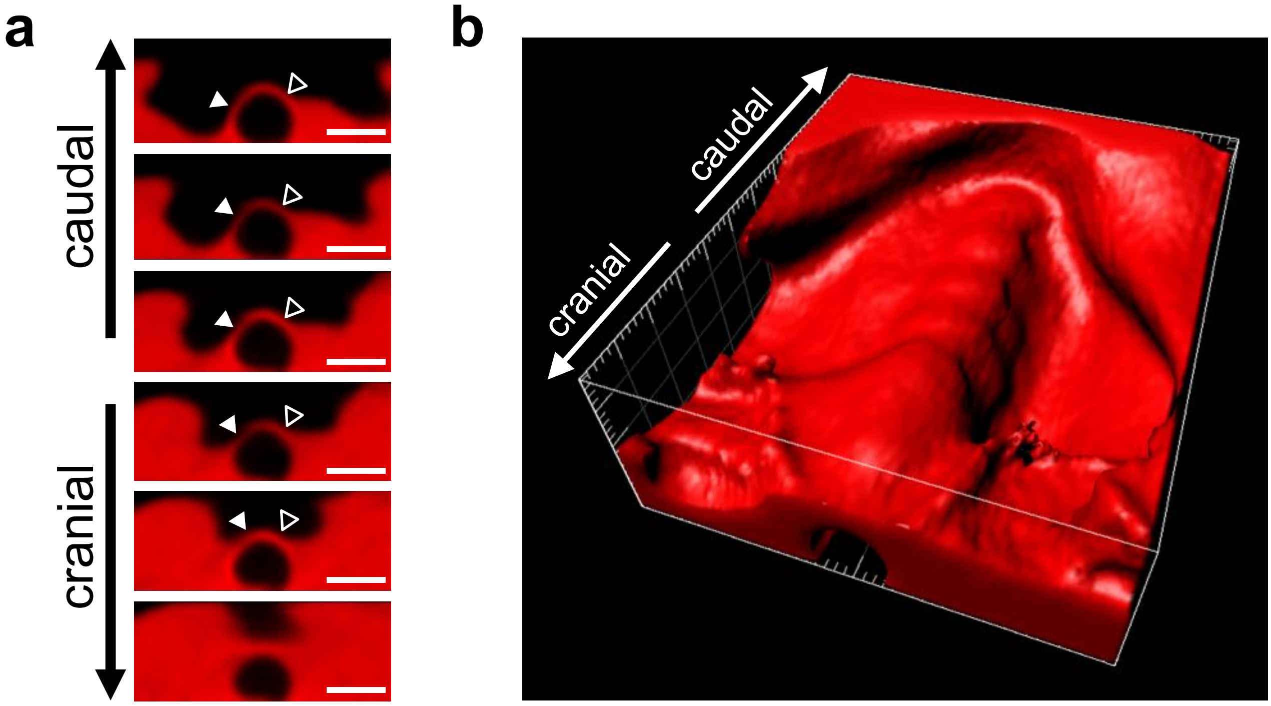

Supplementary Figure 2: 3D reconstruction of tracheal micro-anatomy using micro-CT. (**a**) Serial cross-sectional images of the surgically exposed trachea (the distance between each section is approximately 400 µm). Arrows indicate the left (filled arrow) and right (empty arrow) sides of the trachea (bar=1mm). (**b**) 3D surface-rendered image of the trachea and surrounding tissues, created from the CT-scan dataset shown in (**a**).

Supplementary Figure 3

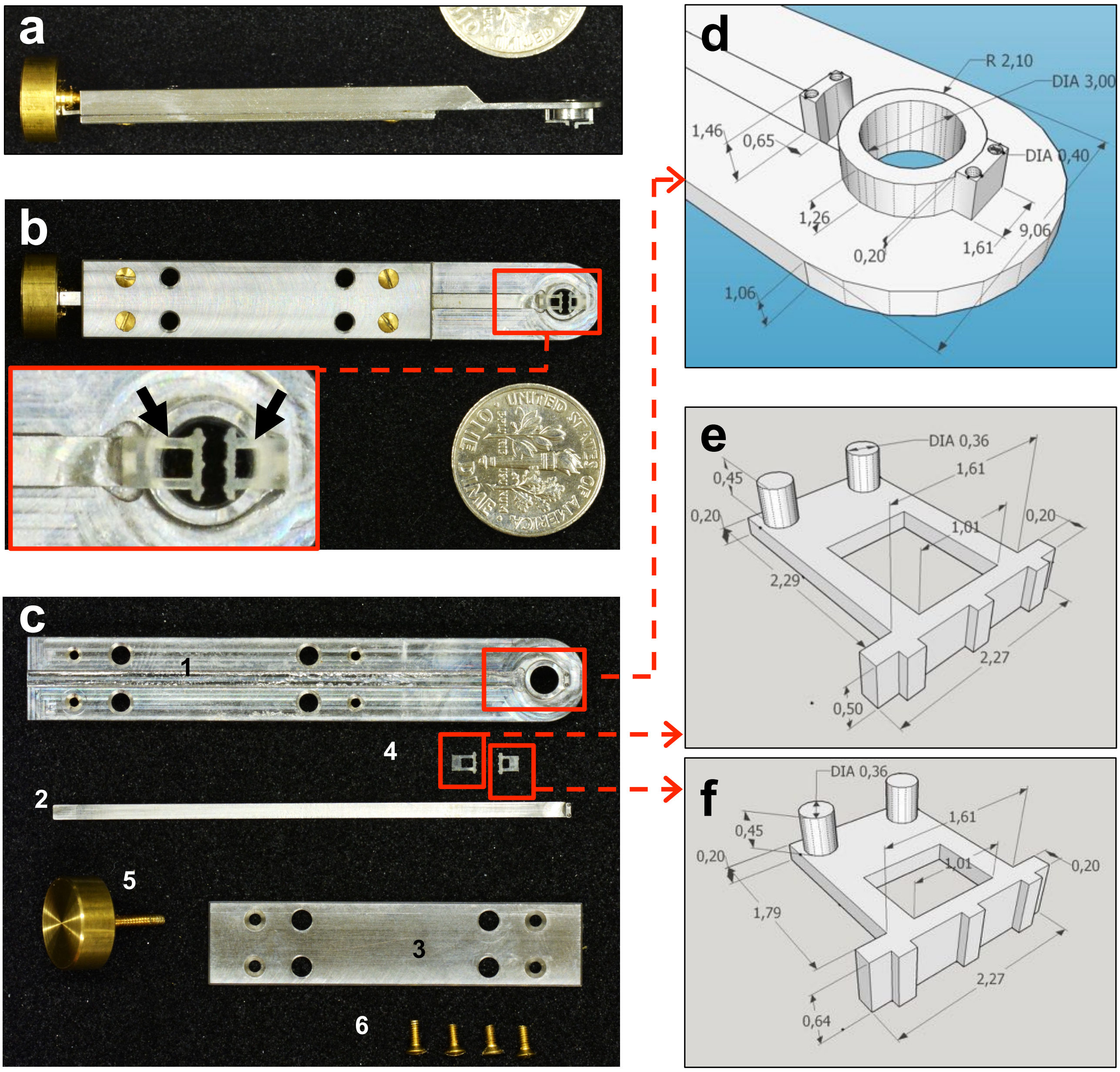

Supplementary Figure 3: Precision parts of the intubation-free tracheal "imaging window" built by CNC milling or 3D printing. (a,b): Assembled device in front-view (**a**) and bottom-view (**b**). Dime was inserted to indicate relative dimensions. In (**b**), insert shows the window area with the 3D-printed lateral supporting parts (arrows). (**c**) Separate parts of the device after disassembly: CNC-milled "main body" (1), adjustable "slider" (2) and cover plate (3); 3D-printed lateral supporting parts (4); adjustment screw (5); screws for assembly (6). (**d-f**) main dimensions of the window (**d**), left (**e**) and right (**f**) lateral supporting parts (all in mm).

Supplementary Figure 4

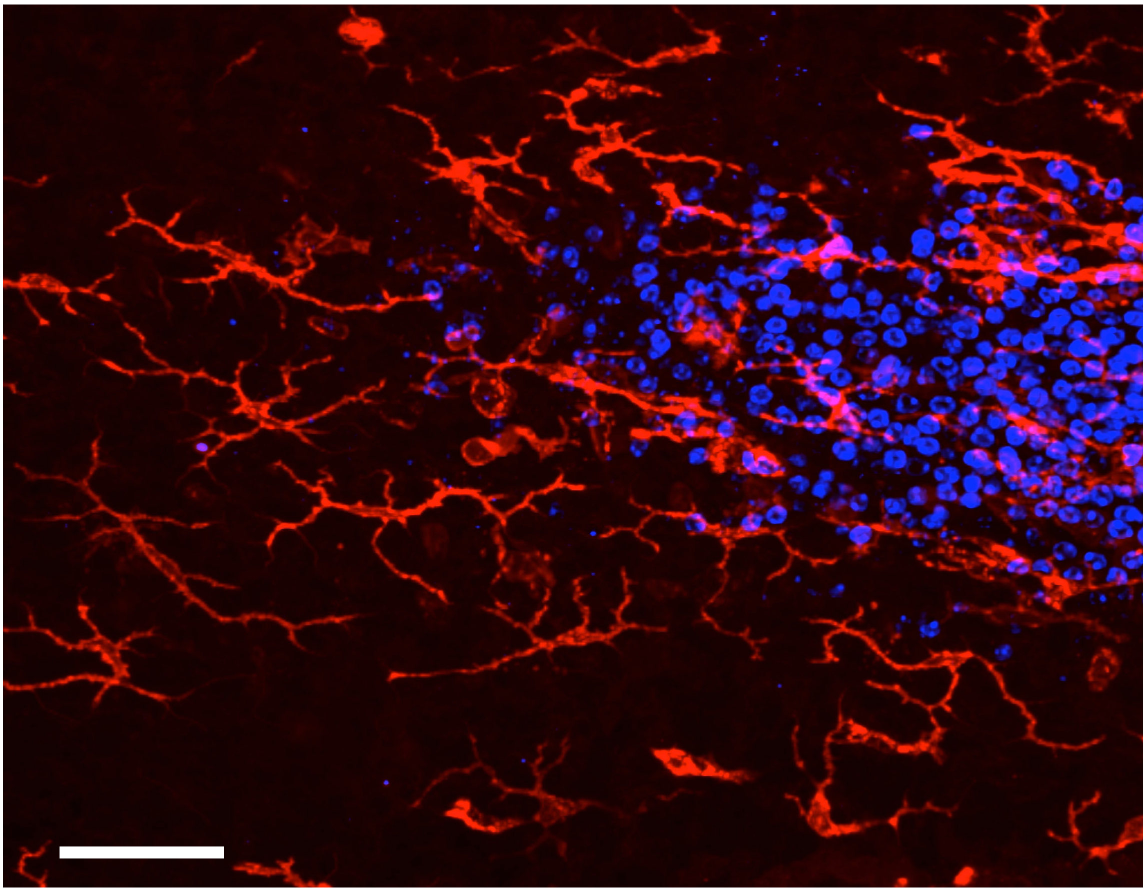

Supplementary Figure 4: The tracheal IE-DC network. Whole-mount immunostaining of a perfusion-fixed trachea for MHC-II (red) to reveal IE-DCs with a prototypical morphology including several dendritic extensions. Nuclei of epithelial cells were revealed by To-Pro-3 staining (blue). Maximum-intensity projection of a confocal Z-stack (bar=50 µm).

Supplementary Video Legends

Supplementary Video 1: Lack of tissue inflammation and vascular leakage in the intubation-free setup. Visualizing blood vessels and the surrounding tissue in the trachea of a CAG-ECFP mouse. 155 kDa TRITC-dextran was i.v. injected prior to image acquisition. Time is shown as h:mm:ss.

Supplementary Video 2: Lack of tissue inflammation and vascular leakage in the intubation-free setup. Visualizing blood vessels and the surrounding tissue in the trachea of a CAG-DsRed mouse. 2,000 kDa FITC-dextran was i.v. injected prior to image acquisition. Time is shown as h:mm:ss.

Supplementary Video 3: Tracheal intubation causes pronounced inflammation and vascular leakage. Visualizing blood vessels and the surrounding tissue in the trachea of an intubated CAG-ECFP mouse. 155 kDa TRITC-dextran was i.v. injected prior to image acquisition. Time is shown as h:mm:ss.

Supplementary Video 4: Visualizing tracheal IE-DC probing activity in a CD11c-EYFP mouse using the intubation-free setup. CD11c-EYFP$^+$ DCs (yellow) are shown together with second-harmonic generation (SHG) signals (cyan). White arrows indicate IE-DCs. XY, XZ and YZ representations are shown. Time is shown as hh:mm:ss:mms.

Supplementary Video 5: Formation of abnormally elongated, immotile extensions by IE-DCs in an intubated animal, despite sufficient blood flow. Among CD11c-EYFP$^+$ DCs (yellow), IE-DCs are indicated by white arrows. Qtracker 655 vascular label (cyan) was i.v. injected during image acquisition. Time is shown as hh:mm:ss:mms.

Supplementary Video 6: Intubation causes altered DC probing activity (example 1). Visualizing IE-DCs in the tracheal epithelium CD11c-EYFP mice using the intubation-free vs. the intubated method (IE-DC on the left is one of the cells also shown in Supplementary Video 4). Note the disappearance of IE-DC extensions with the intubated setup. Time is shown as hh:mm:ss:mms.

Supplementary Video 7: Intubation causes altered DC probing activity (example 2). Visualizing IE-DCs in the tracheal epithelium of CD11c-EYFP mice using the intubation-free vs. the intubated method. Note the disappearance of IE-DC extensions with the intubated setup. Time is shown as hh:mm:ss:mms.

Supplementary Video 8: Visualizing IE-DC probing activity in the context of the epithelium (intubation-free setup). A CD11c-EYFP x UBC-TdTomato mouse was used to visualize DCs (yellow) together with the tracheal epithelium (red). Evans blue dye was i.v. injected directly prior to image acquisition to reveal blood vessels (cyan). XY, XZ and YZ representations are shown. Time is shown as hh:mm:ss.

Supplementary Video 9: High-magnification view of an IE-DC from the same dataset as shown in Supplementary Video 8. Arrows show an intensively probing IE-DC. Note that the cellular extensions of the IE-DC remain in the plane of the basement membrane (i.e., do not penetrate the epithelium). XY and YZ representations are shown. Time is shown as hh:mm:ss.

Supplementary Video 10: Visualizing mucociliary clearance of fluorescent beads in the trachea using the intubation-free setup. 0.5 µm fluorescent beads were instilled into a UBC-TdTomato mouse by the o.ph. route. Directly after bead instillation, the trachea was prepared for MP-IVM using the intubation-free approach. Just before image acquisition, Evans blue dye was injected into the tail vein catheter. Movie shows a stream of moving beads/bead aggregates (yellow) on the luminal side of the tracheal epithelium (red). Note the adherence of a few beads to the epithelium after the stream and the lack of dye leakage from blood vessels (cyan). XY, XZ and YZ representations are shown. Time is shown as hh:mm:ss:mms.

Supplementary Video 11: Lack of mucociliary clearance of fluorescent beads in the trachea using the intubated setup. 0.5 µm fluorescent beads were instilled by the o.ph. route into a UBC-TdTomato mouse. Directly after bead instillation, the trachea was prepared for MP-IVM and immobilized using an intubation cannula. Just before image acquisition, Evans blue dye was injected into the tail vein catheter. Movie shows immotile beads/bead aggregates (yellow) on the luminal side of the tracheal epithelium (red). Note the leakage of injected dye from blood vessels (cyan). XY, XZ and YZ representations are shown. Time is shown as hh:mm:ss.

Supplementary Video 12: Video-rate imaging of mucociliary bead transport (example 1). 0.5 µm fluorescent beads were instilled by the o.ph. route into a UBC-TdTomato mouse. Directly after bead instillation, the trachea was prepared for MP-IVM using the intubation-free approach. Video shows the real-time flow of beads/bead aggregates (yellow) together with the apical layer of the epithelium (red). Time is shown as h:mm:ss.

Supplementary Video 13: Video-rate imaging of mucociliary bead transport (example 2). 0.5 µm fluorescent beads were instilled by the o.ph. route into a UBC-TdTomato mouse. Directly after bead instillation, the trachea was prepared for MP-IVM using the intubation-free approach. Video shows the real-time flow of beads/bead aggregates (yellow) together with the apical layer of the epithelium (red). The minor image instability is caused by breathing movements. Time is shown as h:mm:ss.
